# Safety and efficacy of Silodosin as medical expulsive therapy after shock wave lithotripsy in paediatric patients ‍‍with renal stones

**DOI:** 10.1007/s00240-025-01760-x

**Published:** 2025-05-20

**Authors:** Mohammed Lotfi Amer, Mohamed Essam Elkholefy, Heba Dawoud, Mohamed Gaber, Osama Mostafa El-gamal, Tarek Gameel

**Affiliations:** 1 Urology Department, Faculty of Medicine, Tanta University Hospital, Tanta, Egypt; 2 Paediatric Department, Faculty of Medicine, Tanta University Hospital, Tanta, Egypt

**Keywords:** Paediatric renal stones, Shockwave lithotripsy, Medical expulsive therapy, Stone-Free rate

## Abstract

This study was designed to assess the safety and efficacy of Silodosin as a medical expulsive therapy following shockwave lithotripsy (SWL) in paediatric patients with renal stones. In this prospective randomized controlled study conducted at Tanta University Hospital from January 2022 to March 2024, thirty children with a single de novo radiopaque renal pelvic stone less than 2 cm scheduled for SWL were randomized into two equal groups. Group A (*n* = 15) received Silodosin 4 mg once daily after the first SWL session, and Group B (*n* = 15) received a matching placebo. The first dose was administered on the night of the initial SWL session and continued until stone-free status was confirmed, for a maximum of 4 weeks. The stone expulsion time was set as a primary outcome, while the secondary outcomes were one-session stone-free rate (SFR), postoperative pain scores, and Silodosin related adverse events. The results showed that the mean stone expulsion time in group A (11.4 ± 1.8 days) was significantly shorter compared to group B (16.4 ± 1.6 days; *P* < 0.0001). One-session SFR was 86.6% in Silodosin group compared to 73.3% in Placebo group (*P* = 0.6). Pain visual analogue scores were significantly lower in group A (2.31 ± 1.75) than in group B (5.08 ± 2.43; *P* = 0.003). No severe drug-related adverse effects were reported in either group. In conclusion, Silodosin appears to be a safe and effective adjunct to SWL in paediatric patients, significantly reducing stone expulsion time and postoperative pain. Larger studies are needed to confirm these findings.

## Trial registration number

PACTR202503893791588 (retrospectively registered).

## Introduction

Renal stones are becoming increasingly common in children, likely due to factors such as dietary habits, lifestyle changes, and environmental conditions [27, 31, 33]. They can lead to significant health issues, including recurrent UTIs, pain, haematuria, and potential renal function deterioration, with urging need for effective management strategies [6].

Shock wave lithotripsy (SWL) remains a standard non-surgical approach for managing renal calculi less than 2 cm in size, relying on shock waves to fragment stones into passable pieces [5, 30]. However, residual stone fragments can remain, necessitating additional interventions to prevent complications such as obstruction, infection, and recurrence [25]. The first use of SWL in children was reported by Newman et al. in 1986 [20]. Since this date, SWL has been reported as one of the pivotal options of treatment of pediatric stones with high success rate ranging from 70 to 96% and acceptable safety profile [9].

Medical expulsive therapy (MET), often used alongside SWL, aims to facilitate the passage of stone fragments. Alpha 1 adrenoreceptors are highly distributed in the lower part of ureter and blockage of these receptors improves stone passage by inhibition of uncoordinated contractions and prevention of ureteric muscle spasm [29]. Alpha-blockers, like Tamsulosin, have been shown to enhance stone clearance rates and shorten expulsion time by relaxing the ureteric smooth muscle [14, 24]. However, their use in paediatric patients remains unexplored, raising concerns regarding safety and efficacy [15].

The safety of selective alpha antagonists in pediatrics especially Tamsulosin has been reported by various studies [2,19,32].In 2012, FDA approved the safety of Tamsulosin in children with daily dose of 0.2 mg for children under 5 years old and a conventional dose of 0.4 mg daily for older children [13].

Silodosin, a selective α1A-adrenoceptor antagonist, targets the lower urinary tract’s smooth muscle, potentially offering fewer systemic side effects than non-selective alpha-blockers, making it a promising option for paediatric patients [1, 17]. By blocking α1A adrenergic receptors -primarily found in the prostate and bladder neck- Silodosin facilitates muscle relaxation and stone passage [7, 16, 23, 36]. While effective in adults, its use in paediatric patients is not fully explored. This study aims to evaluate Silodosin’s efficacy and safety as an adjunct therapy to SWL in children with renal pelvic stones.

## Methods

### Study design and setting

This study was a prospective, double-blind, randomized controlled trial conducted at the Department of Urology, Tanta University Hospital, from January 2022 to March 2024. Ethical approval (*approval number 32649/10/18*) was obtained, and informed consent was signed by the guardians of all participating children after thorough discussion about the benefits and the possible undesirable effects of the off-label use of Silodosin. The study was retrospectively registered in the Pan African Clinical Trials Registry *(Trial registration number PACTR202503893791588).*

### Study population

The study included paediatric patients under 18 years old with newly diagnosed single radiopaque renal pelvic stone scheduled for SWL.We excluded patients with a solitary kidney,congenital or acquired renal or ureteral abnormalities, significant hydronephrosis (as per Onen’s criteria) [21],presence of a JJ stent, active UTIs, or impaired renal function (eGFR < 60 mL/min/1.73 m²).

**Fig. 1:**
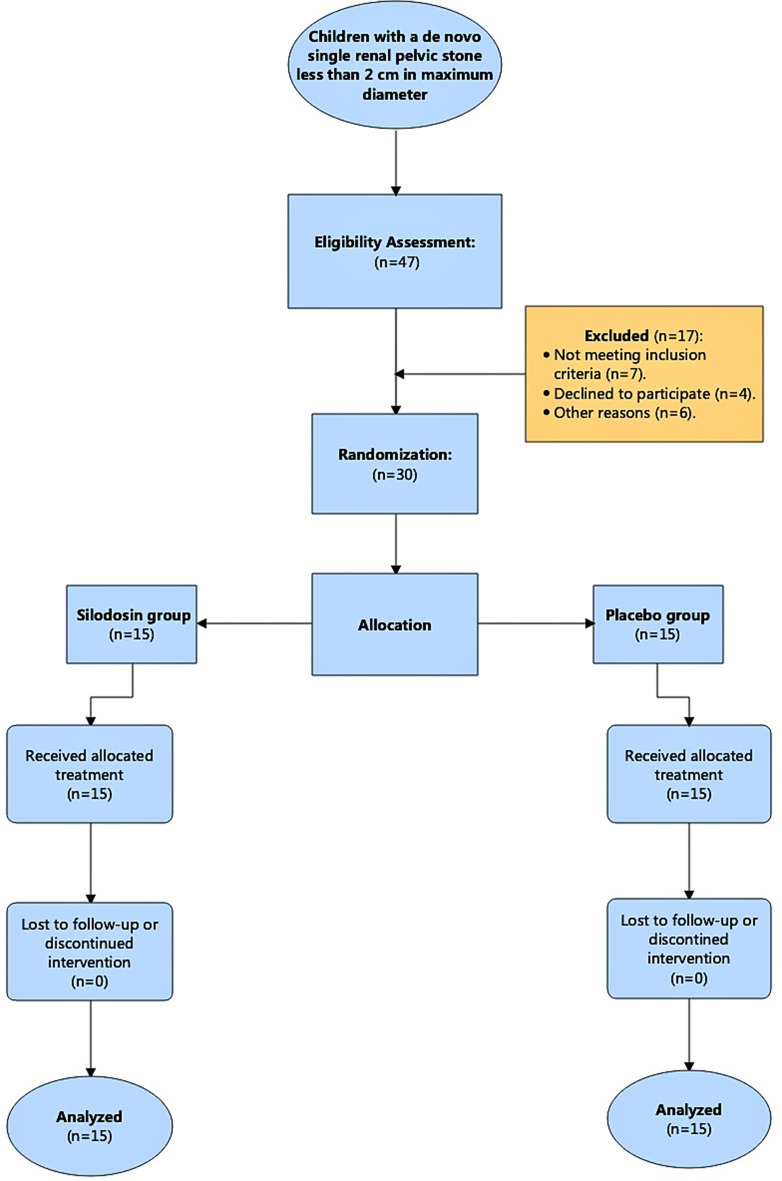
Flowchart of the study recruitment process

Thirty paediatric patients were enrolled, randomized and divided using computer randomization software [26] into two equal groups (15 in each group) before the first SWL session **(**Fig. 1**).**

1. Silodosin group (Group A):Patients received 4 mg Silodosin (Sildocare 4 mg ^®^, Andalous Pharma Company) as a single bedtime dose in a capsule form. The first dose was administered on the night of the initial SWL session and continued until stone-free status was confirmed, for a maximum of 4 weeks. For patients who had difficulty swallowing capsules, the contents were emptied and mixed with water, juice, or yogurt.
2. Placebo Group (Group B):Patients received an identical placebo capsule as a single bedtime dose, ensuring the blinding of both participants and investigators.

### Sample size calculation

The sample size calculation for this study was based on the primary endpoint, stone expulsion time for small ureteric stone fragments, comparing Silodosin to standard care following SWL for renal pelvic stones. Based on previous research, without MET after SWL, the passage of small stone fragments might long for 2–4 weeks [4, 22]. We hypothesized that Silodosin use could reduce this time to 1–2 weeks, based on similar studies on ureteric stones [10], leading to a mean expulsion time of 14 days in the Silodosin group versus 21 days in the control group.

Based on a two-sample t-test for continuous variables, with a two-sided alpha of 0.05 and 80% statistical power, the calculated minimum sample size needed for each group was 13 participants, resulting in a total of 26 patients. To account for potential dropouts and protocol deviations, we recruited 15 patients in each group with a total sample size of 30 patients.

All participants underwent thorough preoperative evaluations, including a thorough medical evaluation, complete blood count, coagulation profile, renal function assessment, and urinalysis. Imaging studies (Kidney Ureter Bladder X-ray films (KUB), ultrasonography, non-contrast computed tomography scans of abdomen and pelvis NCCT) were conducted to confirm stone size and location. UTIs were treated with antibiotics prior to the procedure. Preoperative fasting was required (6 h for solids, 2 h for fluids).

### Anaesthesia and sedation

Standardized anaesthesia protocols were used. General Anaesthesia (GA) was induced in all children less than 10 years or uncooperative children, using propofol (1–2 mg/kg) and maintained with sevoflurane (2–3%). Pain control measures included administration of paracetamol (15 mg/kg) or ibuprofen, with fentanyl (1–2 µg/kg) as needed. Sedation was induced using Midazolam (0.1–0.2 mg/kg) when GA was not required. Continuous monitoring of vital signs was implemented.

### Shock wave lithotripsy procedure

SWL was performed using the Dornier Compact Delta II Lithotripter, under fluoroscopic guidance. The procedure involved 1,500 to 3,000 shocks per session, with power settings adjusted based on patient tolerance -if sedated- with a range of settings (14–16 kV, 60–80 shocks per minute).

### Patient instructions

Patients and guardians were provided with standardized instructions to enhance compliance and data accuracy:

1. The patients received dietary guidance to promote hydration (at least 2 L of water daily) and engage in regular physical activity. Adherence was tracked through diaries.
2. Pain Management Protocol:Diclofenac (1–2 mg/kg) was administered orally or rectally guided by the severity of pain episodes. If pain was not responding to regular analgesia, in hospital admission was requested.
3. Symptom Diary: Record episodes of pain, analgesic use, and stone expulsion events.
4. Monitoring Stone Passage: Use a potty for voiding to monitor and collect expelled stone fragments for chemical analysis.
5. Haematuria Monitoring:‍‍ Observe urine for blood and report the duration until it clears.
6. Emergency Protocol:‍‍ Seek immediate medical attention for fever, persistent pain, or prolonged haematuria.

### Post-procedural monitoring and Follow-Up

All patients were subjected to regular follow up visits twice weekly; however, they were monitored radiologically at two time points (2 and 4 weeks of starting the treatment) using KUB radiographs and ultrasonography to assess stone clearance. Stone free status was defined as no stones or < 4 mm fragments in radiological studies after 2 or 4 weeks of SWL session. Presence of stone fragments (> 4 mm) after 4 weeks was an indication for another SWL session.

Stone expulsion time was recorded as the number of days from the first SWL session until the last stone fragment was visually confirmed to have passed. This was confirmed by absence of ureteric stone fragments during radiological assessments at the checkpoints 2 and 4 weeks.

Follow up assessments included measurement of blood pressure and pulse rate in pediatric reception. The cuff of the used sphygmomanometer should be appropriate to arm size as its width must be 40% of the arm circumference at mid arm point between acromion and olecranon. The recommended size of cuff in infant is 6 × 12 cm and the recommended size in older children is 9 × 18 cm. The child should be rested and relaxed with his right arm at the level of the heart.

For both study groups, data collection included stone expulsion time and rate, number and intensity of pain episodes, analgesic consumption, and any adverse events. Serious complications were immediately reported to the principal investigator and the institutional ethics committee.

Pain severity was assessed using the Visual Analogue Scale (VAS), with scores provided by the parents [35]. Any instance of abdominal pain, whether typical of renal colic or atypical, that required administration of analgesics during the study duration was defined as a pain episode. Following stone expulsion, the patients were referred to paediatric department for dedicated metabolic evaluations and tailored management strategies.

### Statistical analysis

Collected data included pain scores, analgesic usage, stone expulsion time, one session SFR, duration of haematuria, and adverse events. Baseline characteristics, stone expulsion time, pain scores, one session stone clearance rates, and adverse events were evaluated using suitable statistical assessments, such as descriptive statistics, independent t-tests, Chi-square tests, and Fisher’s exact test where applicable. Data analysis was performed with SPSS version 27, considering a p-value < 0.05 as statistically significant and presenting results with 95% confidence intervals. To minimize bias and enhance internal validity, this study employed blinding and allocation concealment.

## Results

### Patients demographic data

Thirty children diagnosed with de novo renal pelvic stones were included in the current study, with comparable baseline demographic data **(**Table 1**)**. The mean age was 8.6 ± 3.2 years in group A, and 8.9 ± 3.2 years for group B. A higher percentage of males were noticed in group B (80%) compared to group A (53.3%). Right-sided kidney stones were more common in both groups. Symptoms such as nonspecific abdominal pain, haematuria, loin pain, and dysuria were comparable in both groups **(**Table 1**)**.

**Table 1:** Patients’ demographic data and stones’ characteristics

Parameter	Silodosin Group	Placebo Group	Statistical test	*P* value
**Age (years) ± SD**	8.6 ± 3.2	8.9 ± 3.2	t = 0.359	*P* = 0.46
**Male n (%)**	8 (53.3)	12 (80)	X²=2.4	*P* = 0.121
**Female n (%)**	7(46.7)	3(20)		
**Stone size** (cm)	1.31 ± 0.19	1.21 ± 0.2	t = 1.829	*P* = 0.187
**Hounsfield Unit (HU)** m± SD	836.67 ± 106.01	821.33 ± 111.03	t = 0.15	*P* = 0.702
**Stone side** Right kidney n (%) Left kidney n (%)	11(73.3)	8(53.3)	X²=0.833	*P* = 0.361
	4(26.7)	7(46.7)		
**Complaint at presentation**				
Non-specific abdominal pain	7(46.7)	11(73.3)	2.222	*P* = 0.136
Haematuria	6(40)	4(26.7)	0.6	*P* = 0.43
Loin pain	5(33.3)	4(26.7)	0.159	*P* = 0.69
Dysuria	3(20)	2(13.3)	0.269	*P* = 0.604

### procedural data and stone outcomes

General anaesthesia was used in 43.3% of cases, while 56.6% received sedation. Both groups underwent similar numbers of shock waves during SWL, with no significant differences in procedural characteristics **(**Table 2**)**. One session SFR was slightly higher in group A (86.6%) compared to group B (73.3%), but without statistically significant difference (*P* = 0.65). The Cramér’s V for this comparison was 0.083, indicating a small effect size and suggesting a weak association between treatment group and stone clearance.

**Table 2:** Procedure technical details and outcomes

Parameter	Silodosin Group	Placebo Group	Statistical test	*P* value
**General Anaesthesia** n (%)	8(53.3)	5(33.3)	X²=3.333	*P* = 0.068
**Number of Shocks** m ± SD	2066.67 ± 231.97	2126.67 ± 357.50	t = 0.297	*P* = 0.59
**Frequency of shock waves** m ± SD	71.33 ± 8.34	77 ± 7.75	3.719	*P* = 0.064
**Procedure Time (minutes)** ± SD	30 ± 3.78	30.67 ± 4.17	t = 0.211	*P* = 0.65
**One session SFR** n (%)	13(86.7)	11(73.3)	X²=0.833	*P* = 0.6
**Stone Expulsion Time (days)** m ± SD	11.4 ± 1.8	16.4 ± 1.6	t = 49.545	*p* < 0.0001
**Complications after SWL**				
Mild visible haematuria n (%)	6(40)	5(33.3)	X²=0.142	*P* = 0.705
Significant Pain n (%)	6(40)	13(86.7)	X²=7.032	*P* = 0.008
Hypotension Fever Subcapsular hematoma	0	0	-	*-*
**Drug related adverse events**				
Gastrointestinal Symptoms n (%)	3(20)	0	-	-
Headache/Dizziness n (%)	2(13.3)	0	-	-
**Post procedure Pain Score** m ± SD	2.31 ± 1.75	5.08 ± 2.43	t = 11.109	*P* = 0.003
**Analgesic Need** n (%)	6(40)	13(86.7)	X²=7.032	*P* = 0.008
**Need for Analgesic Injection** n (%)	2(13.3)	7(46.7)	X²=3.968	*P* = 0.046

On the contrary, the mean stone expulsion time was significantly shorter in group A (11.4 ± 1.8 days) compared to group B (16.4 ± 1.6 days; *P* < 0.0001) **(**Table 2**)**. The Cohen’s d for this difference was − 2.94, indicating a very large effect size. This suggests that Silodosin accelerates the stone expulsion process by almost 3 standard deviations, highlighting its substantial clinical benefit in reducing expulsion time. Therefore, while Silodosin improved stone expulsion time, it did not significantly influence the overall clearance of stones after one session of SWL compared to placebo.

### Complications and pain management

Only minor complications (Clavien-Dindo grade 1) were reported in 56.6% of patients, primarily mild visible haematuria and postoperative pain. Haematuria occurred in 40% of the children in group A and 33.3% of the patients in group B, with no significant difference. Postoperative pain scores were significantly lower in the group A (2.31 ± 1.75) compared to group B (5.08 ± 2.43; *p* = 0.003). The analgesics use was also significantly reduced in the group A (40% vs. 86.7% in group B; *p* = 0.008) **(**Table 2**)**. The Cohen’s d for this comparison was − 1.31, indicating a large effect size. This demonstrates that Silodosin effectively reduces postoperative pain, with a clinically meaningful difference of more than 1.3 standard deviations between the groups.

Six children (20%); 2 in group A (6.6%) and 4 in group B (13.3%) who needed a second SWL session. All their fragments were inside the kidneys without any ureteric stones detected. Five of these patients were stone free after the 2nd SWL session and only one patient in group B needed a 3rd SWL session and became stone free after that. None of the patients required an auxiliary procedure other than SWL.

### drug related adverse events and metabolic findings

Adverse events associated with Silodosin were mild, with gastrointestinal symptoms (nausea and vomiting) reported in 20% of patients, resolving without intervention. No episodes of hypotension were noted, however two patients experienced transient headache and dizziness episodes which didn’t affect their compliance to medication **(**Table 2**)**.

## Discussion

In the present study, we examined the effectiveness of Silodosin as a MET following shock wave lithotripsy (SWL) for children with a de novo single renal pelvic stone smaller than 2 cm. All stones were radiopaque, with a mean size of 1.2 ± 0.3 cm and a mean stone density of 829 ± 106.9 HU. Radiolucent stones were excluded due to the lack of ultrasonic localization capabilities in our equipment.

Regarding anaesthesia, GA was administered to 10 children (33.3%) aged 10 years or younger, and to 3 children (10%) over the age of 10 who exhibited uncooperative behaviour. Sedation was used for 17 children (56.6%). These findings slightly diverge from the study by Jee et al. [18], where GA was employed for patients aged 7 years or younger, while those over 7 years were managed with sedo-analgesia [18]. Additionally, their study reported that nearly third of the patients older than 7 years required conversion to general anaesthesia due to poor cooperation. In contrast, Shahat et al. used GA for all patients, explaining this decision by the younger ages of their study cohort (4.04 ± 2.5) years [28].

In terms of shock wave parameters, the number of shocks ranged from 1500 to 2500, with a force between 12 V and 16 V, and a frequency of 60 to 85 shocks per minute. These parameters are consistent with those reported by Shahat et al., Jee et al., and João Paulo da Cunha Lima et al. The variations observed in shock wave count, rate, and force can be attributed to factors such as patient cooperation, stone size, and stone density [8, 18, 28].

Before the first SWL session, the patients were randomly assigned to either the Silodosin group or the placebo group. Our results showed a marginally higher one-session SFR in the Silodosin group (86.7%) compared to the placebo group (73.3%). However, this difference did not reach statistical significance (*P* = 0.6), and the effect size was minimal, as indicated by a Cramér’s V of 0. 083.This finding suggests that while Silodosin may assist in the expulsion of stone fragments, it does not significantly impact the overall clearance of stones. One potential explanation for this could be the variability in stone fragmentation following SWL, which may influence the expulsion process.

This result is consistent with the study by Elgalaly et al., which also reported no significant difference in treatment efficacy between silodosin and placebo for managing distal ureteric stones in paediatric patients [10]. Similarly, Aydogdu et al. reported comparable findings in their study on doxazosin for distal ureteric stones [3]. Conversely, Fahmy et al. found a significant difference in stone clearance between Silodosin (78.5%) and placebo groups (53.3%), as well as between Tamsulosin (66.6%) and placebo [12]. Additionally, Mokhless et al. reported a significant enhancement in SFR among children receiving tamsulosin compared to those given placebo. Similarly, Erturhan et al. demonstrated that doxazosin was more effective than ibuprofen in promoting stone expulsion in paediatric patients with distal ureteric stones [11, 19].

The mean stone expulsion time was noticeably shorter in the Silodosin group (*P* < 0.0001). This result agrees with findings by Elgalaly et al. and Fahmy et al., both of whom demonstrated that Silodosin significantly shortened the expulsion time for distal ureteric stones in children [10, 12]. Additionally, Onur Telli et al. observed that doxazosin, as an adjunctive therapy after SWL for renal stones in children, reduced expulsion time more effectively than a watchful waiting approach [34].

Pain scores and the requirement for analgesia during stone expulsion were also evaluated. The Silodosin group reported significantly lower pain scores compared to the placebo group (*P* = 0.003). Similarly, the requirement for analgesics was notably greater in the placebo group compared to those receiving silodosin (*P* = 0.008). These results are in agreement with previous findings by Elgalaly et al. and Fahmy et al., who similarly reported lower pain levels and decreased analgesic consumption among paediatric patients treated with silodosin during fragment stones passage [10, 12].

The safety profile of selective alpha-blockers in paediatric patients has been reported in several studies [3, 11]. Doxazosin has been used without significant complications in various studies, and the FDA approved Tamsulosin for use in children in 2012 [13]. In the current study, Silodosin was generally well tolerated, with side effects observed in only five patients, manifesting as mild gastrointestinal upset in 3 patients, including nausea and vomiting, while 2 patients complained from some episodic headache and dizziness. No patients discontinued treatment due to side effects. These results are consistent with the findings of Elgalaly et al. and Fahmy et al., who also demonstrated that Silodosin has a favourable safety and tolerability profile in children [10, 12].

Overall, this study demonstrated that Silodosin, when used as a MET following SWL in paediatric patients with renal stones, significantly reduced stone expulsion time and postoperative pain compared to placebo without significant adverse events.

### Limitations of the study

First, the sample size of only 30 patients, while sufficient for detecting large variability effects, indeed limits the ability to generalize the results. Second, we only included index patients with favourably positioned stones in the renal pelvis. Therefore, the results can’t be generalized to cover all SWL scenarios where other factors can affect the success rate. Additionally, expanding the inclusion criteria to include patients with different types of stones (e.g., non-radiopaque stones) and comorbid conditions could provide a more comprehensive understanding of Silodosin’s role in renal stone management across diverse clinical scenarios.

Future studies with larger, multi-centre cohorts are essential to confirm the efficacy and safety of Silodosin in a broader paediatric population focusing on its use in different types of renal stones or with variations in SWL protocols to better understand its impact on stone clearance. Additionally, comparative trials with other alpha-blockers, such as Tamsulosin, would help elucidate whether Silodosin offers superior efficacy in paediatric patients.

## Conclusion

This study demonstrates that Silodosin could be a safe and effective adjunct therapy following SWL in paediatric patients with renal stones. It significantly reduces stone expulsion time, alleviates postoperative pain, and decreases the need for analgesics compared to placebo. While the results are promising, the study’s small sample size and the lack of significant findings in stone clearance highlight the need for further research. Larger, multi-centre trials are warranted to confirm these findings and influence clinical guidelines.

## Data Availability

No datasets were generated or analysed during the current study.
